# Effect of green tea extracts on oxaliplatin-induced peripheral neuropathy in rats

**DOI:** 10.1186/1472-6882-12-124

**Published:** 2012-08-15

**Authors:** Jung Soo Lee, Yoon Tae Kim, Eun Kyoung Jeon, Hye Sung Won, Young-Seok Cho, Yoon Ho Ko

**Affiliations:** 1 Department of Rehabilitation Medicine, Uijeongbu St. Mary's hospital, The Catholic University of Korea, Uijeongbu, South Korea; 2 Division of Oncology, Department of Internal Medicine, Uijeongbu St. Mary's hospital, The Catholic University of Korea, 65-1, Geumo-dong, Uijeongbu- si, 480-717, South Korea; 3 Division of Gastroenterology, Department of Internal Medicine, Uijeongbu St. Mary's hospital, The Catholic University of Korea, Uijeongbu, South Korea

**Keywords:** Oxaliplatin, Peripheral neuropathy, Green tea extract, Antioxidant

## Abstract

**Background:**

A common side effect of oxaliplatin is peripheral neurotoxicity. Oxidative stress to dorsal root ganglion (DRG) may be one of important pathogenic mechanisms. Green tea contains four polyphenol catechins, which are known to be potent antioxidants. The present work is aimed to determine whether green tea extracts have neuroproective or palliative effects on neurotoxicity symptoms induced by oxaliplatin.

**Methods:**

We conducted behavioral tests including sensory and thermal thresholds, an electrophysiological study, and TUNEL staining to assess neurotoxicity during the experimental period using animal models.

**Results:**

A total of 14 adult rats were randomly allocated into two groups. Oxaliplatin (4 mg/kg) with or without green tea (300 mg/kg orally once daily) was administered intraperitoneally twice per week for 6 weeks. At 4 and 6 weeks after oxaliplatin administration, sensory threshold values were significantly decreased and at 6 weeks after oxaliplatin administration, thermal threshold values were significantly increased in oxaliplatin-treated rats compared with those in rat treated with oxaliplatin and green tea extracts. The electrophysiological assessment, including sensory nerve conduction and H-reflex-related sensory nerve conduction velocity, revealed no significant changes in the two groups. TUNEL staining showed no significant difference in the number of apoptotic-featured cells between the two experimental groups in the DRG or peripheral nerves, but the number of apoptotic-featured cells in DRG was higher than that in sciatic nerves within each group.

**Conclusions:**

Green tea extracts may be a useful adjuvant to alleviate sensory symptoms after oxaliplatin administration, such as allodynia, but did not prevent morphometric or electrophysiological alterations induced by oxaliplatin.

## Background

Neurotoxicity is a common adverse effect associated with antineoplastic agents. Of these agents, oxaliplatin has been commonly used in clinical practice to treat various cancers of the gastrointestinal tract. A common and dose-limiting side effect of oxaliplatin is peripheral neurotoxicity (PN), which is known to be cumulative [1]. At cumulative doses that reach 800 mg/m^2^, the occurrence of neurotoxicity is highly likely. A total neuropathy score ≥ grade 3 PN occurs in 15% of cases after cumulative doses of 750-850 mg/m^2^ and in 50% of cases after a total dose of 1170 mg/m^2^[2].

Oxaliplatin treatment induces an acute neurotoxicity, characterized by a rapid onset of cold-induced distal dysesthesia and a chronic sensory peripheral neuropathy [1]. The clinical similarity between the acute oxaliplatin-induced PN symptoms, such as transient paresthesia and dysesthesia in the perioral area, and conditions caused by impairment of neuronal ion channels suggests that oxaliplatin interacts with ion channels located in the cellular membrane [3] or induces alteration in voltage-gated sodium (Na^+^) channel function [4]. On the other hand, the chronic, sensory, oxaliplatin-induced PN may be induced by decreased cellular metabolism and axoplasmatic transport secondary to the accumulation of platinum compounds in dorsal root ganglia (DRG) cells [5], by the impairment of cellular mitochondrial oxygen consumption [6], or by the prolonged activation of voltage-gated Na^+^ channels, which induce cellular stress in sensory nerve cells via excess Ca^2+^ influx [7].

Antineoplastic agents are known to produce oxidative stress, reduce the total radical-trapping capacity of blood plasma, and decrease plasma levels of antioxidants [8]. Reactive oxygen species modulate Na^+^ channel activity [9], a class of ion channels that has been suggested to enhance nociceptor activity in patients with acute oxaliplatin-induced PN [10]. Moreover, the evidence that DRG oxidative stress may be an important pathogenetic mechanism for chronic toxicity is increasing [11].

Dose-limiting neuropathies may require clinicians to adjust chemotherapeutic doses. An ideal approach is to prevent or minimize neuropathy symptoms and not to reduce the efficacy of chemotherapeutic agents against tumors. Though an effective treatment of established chemotherapy-induced PN has not been found, many prevention and treatment strategies have been suggested, including amifostine, acetyl-L-carnitine, and antioxidants, such as vitamin E, vitamin C, β-carotene, and glutathione [12]. Green tea contains four polyphenol catechins: gallocatechin, epigallocatechin, epicatechin, and epigallocatechin-3-gallate (EGCG) [13]. Because polyphenol catechins are known to be potent antioxidants, catechins have been investigated for their ability to prevent cancer development and chemotherapy-induced toxicities [14]. Platinum antineoplastic agents have been shown to induce a fall in plasma antioxidant levels, due to oxidative stress in human studies, and supplementation with antioxidants has shown a protective effect against cisplatin-induced renal toxicity and ototoxicity in animal studies [15]. Thus, based on the above, we conducted an animal experiment to determine whether green tea extracts have neuroproective or palliative effects on neurotoxicity symptoms induced by oxaliplatin.

## Methods

### Animals

Experiments were performed on 301 ± 7 g adult male Sprague-Dawley rats. Animals were housed 3 per cage in a temperature- and humidity-controlled environment under a 12/12-h light/dark cycle. Food and water were freely available. All experimental procedures were approved by the Animal Experimental Committee of the Catholic University of Korea. All efforts were made to minimize the number of animals used and their suffering.

### Experimental design and protocol

In total, 14 animals were randomly allocated into two groups of seven animals. In Group I (*n* = 7), oxaliplatin (4 mg/kg) mixed with 0.9% lactose was administered intraperitoneally twice per week for 6 weeks. In Group II (*n* = 7), in addition to oxaliplatin (4 mg/kg), green tea extracts (Zhejiang Shaoxing Dongling Health Food Co., Ltd; Zhejiang, China), containing 60 mg/kg of EGCG, was administered orally once daily for 6 weeks. We measured weights weekly and conducted behavioral and electrophysiological tests before oxaliplatin administration, at 2, 4, and 6 weeks on all experimental animals. At 6 weeks, DRG and sciatic nerves were harvested from all experimental animals.

### Behavioral studies

#### Assessment of sensory threshold values in hind paws

This test was performed only during the daytime portion of the circadian cycle. Rats were individually placed in a cage with a wire-mesh bottom. They were allowed approximately 15 min for behavioral accommodation, which ended when cage exploration and major grooming activities ceased. We tested the mid-plantar portion of only the right hind paw before the electrophysiological test to avoid the traumatic effect of the insertion of needle electrodes.

The hind paws were first touched with 5.88 monofilament (force gram : 60 gram) of a series of von Frey hairs with logarithmically increasing levels of stiffness (Touch-Test Sensory Evaluators, Stoelting Co., Wood Dale, IL, USA). These were presented perpendicular to the plantar surface with sufficient force to cause slight buckling against the paw, and were then maintained in that position for approximately 6-8 s. Stimuli were presented at intervals of several seconds to allow time for the resolution of the behavioral responses to previous stimuli. A positive response was noted if the tested paw was withdrawn sharply during 5 of the 10 trials. Flinching immediately upon removal of the hair was also considered a positive response if flinching occurred during 5 of the 10 trials. Ambulation was considered an ambiguous response, and the stimulus was repeated in such cases. If a positive response was noted, we selected the one-step lower weight monofilament and, to reconfirm the positive response, the two-step lower weight monofilament was used. If a negative response was noted, we selected the next higher weight monofilament, 6.1 monofilament, but we used 6.1 monofilament only, being afraid of the trauma. The tests were twice repeated on two different daytime by one examiner, and the force grams of monofilaments were averaged.

#### Assessment of thermal threshold values in the tail

A Tail-Flick Analgesia Meter (Harvard Apparatus, Holliston, MA, USA) was used to conduct tail-flick tests within the chamber. A beam of radiant heat (a single, fixed aperture) was focused on the underside of the middle third of the rat’s tail. The latency between exposure to the radiant heat source and movement of the tail away from the focused beam was recorded. The heat intensity was set at 50% of maximal intensity, and maximal exposure to the radiant heat stimulus was 5 s (“cutoff time”) to avoid burn injuries. Since baseline latencies vary between animals, we use the percentage of maximal possible effect (%MPE) as parameter of thermal threshold values. A percentage of maximal possible effect (%MPE) is derived from these measures

(1) $$\%\text{MPE}=\frac{\text{latency}\;\left(\text{s}\right)\;\text{following oxaliplatin administration}-\text{latency}\;\left(\text{s}\right)\;\text{under baseline conditions}}{\text{cutoff time}\;\left(5\;\text{s}\right)-\text{latency}\;\left(\text{s}\right)\;\text{under baseline conditions}}$$

#### Electrophysiology

All measurements were performed by one electrodiagnostic expert while the animals were under general anesthesia (Zoletile, Virvac, France; 30 mg/kg, intraperitoneal injection). Skin temperatures were manipulated using an infrared lamp, and the temperatures of the tail, foot, and leg were checked with an infrared thermometer and ranged between 29 and 30°C.

To assess nerve conduction velocity related to the H-reflex, the left hindlimbs were secured to the long axis of the body at an angle of 30-45°. A monopolar EMG-recording electrode, which served as the active electrode for purposes of recording, was inserted between the left fourth and fifth digits, parallel to the long axis of the left foot. The reference electrode was inserted into the fifth digit of the same foot, and a ground electrode, using a disposable surface electrode, was placed on the tail. The tibial nerve was stimulated through a monopolar cathodal stimulating electrode, which was inserted in the left ankle near the left tibial nerve and in the left hip near the sciatic nerve at the sciatic notch. The anode was placed 5 mm proximal to the cathode. The H-reflex-related sensory nerve conduction velocity (SNCV) was calculated using the latencies of the H-responses at both stimulation points and the distance between the two stimulation points (the sciatic notch and ankle in the gently stretched paw).

To record the sensory nerve action potentials evoked in the tail nerve, we used an antidromic conduction technique. Two subdermal needle electrodes were located in the tail; the active electrode was inserted in the proximal part, and the reference electrode was inserted in the distal part of the tail. The distance between the stimulating and recording electrodes was 7 cm, which was measured on the skin. The nerves were stimulated via monopolar needles by a single pulse delivered by an electrical stimulator with an intensity of 1-2 mA, a duration of 0.1 ms, and a frequency of 1 Hz (Synergy, Medelec, Ltd., Old Woking, England).

### TUNEL method

The TUNEL method was used to observe apoptotic cells induced by chemotherapy using an *in situ* apoptosis detection kit (Takara Bio, Inc., cat. # MK500). Sciatic nerves were harvested 0.5 cm away from the electrode stimulation at the ankle to avoid the possibility of nerve trauma, and four DRG (bilateral fourth and fifth lumbar level) were harvested. Tissues were made into paraffin bocks. Paraffinized nerve tissues were deparaffinized using xylene, 100% ethanol, 90% ethanol, and 80% ethanol, in order, for 5 min, washed with flowing water for 2 min, and immersed in distilled water. After proteinase K (20 μg/mL) was applied, tissues were left at room temperature for 15 min and washed with phosphate-buffered saline. A total of 50 μL of labeling reaction mixture (5 μL TdT enzyme + 45 μL labeling safe buffer) was applied to slides and incubated in a 37°C humidified chamber for 90 min. Apoptotic cells were viewed and counted by at × 200 with a fluorescence microscope at three different points in each tissue sample.

### Statistical analyses

Values are expressed as means ± SD. Normality of distribution was tested with the Shapiro-Wilk test; for thresholds in which the distribution was not within acceptable limits of normality (*P* < 0.05), we used the nonparametric equivalent (the Mann-Whitney test). All tests were one-tailed, and significance was set at *P* < 0.05.

## Results

### Assessment of general toxicity

**Figure 1:**
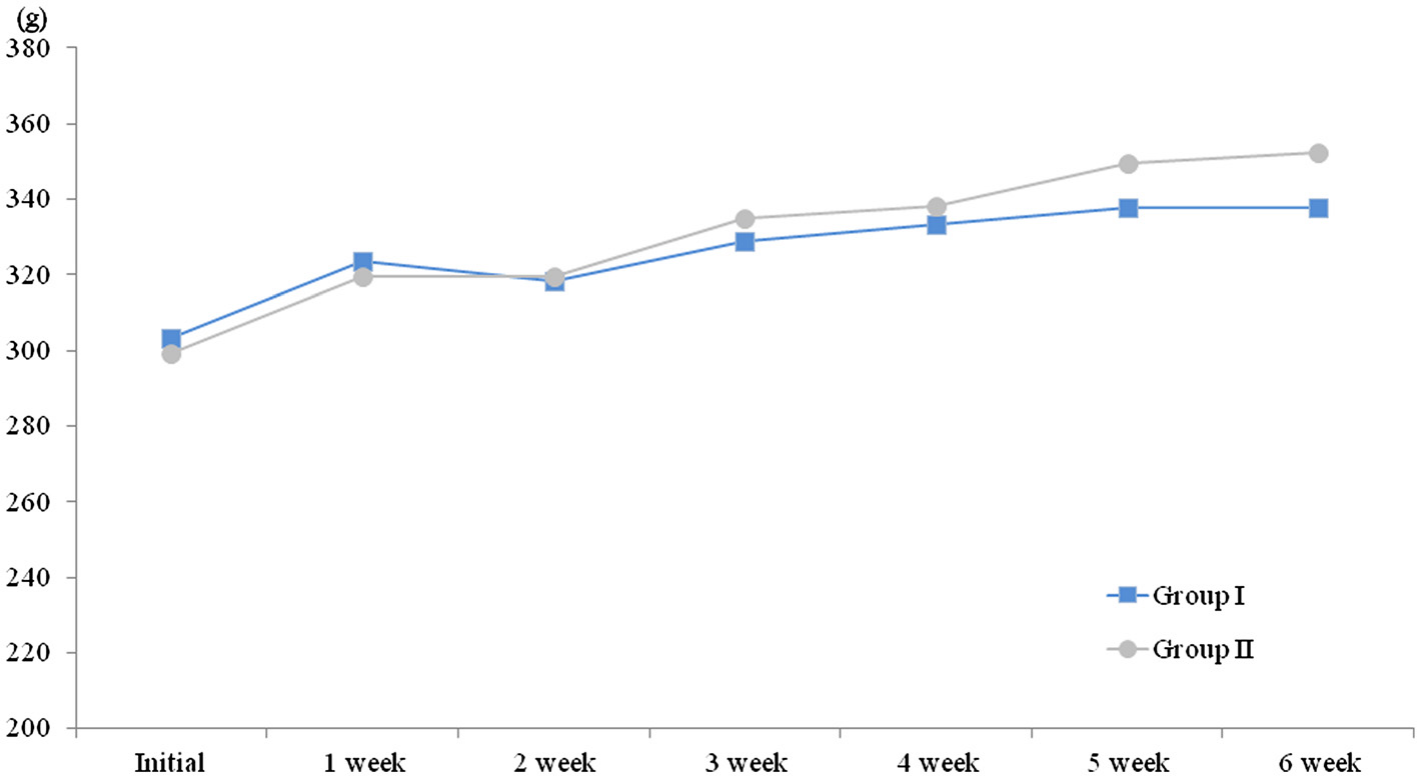
** The assessments of general toxicity.** No changes in weight of rats were observed after oxaliplatin administration between the control and green tea extracts treated groups (p > 0.05).

Before oxaliplatin administration, the weight of Group I was 303 ± 5 g and that of Group II was 299 ± 8 g; these were not statistically significant different (*P* = 0.25). The weights of the rats in Group I increased from 303 ± 5 g to 338 ± 15 g, and the weights of the rats in Group II increased from 299 ± 8 g to 352 ± 20 g during the course of the experiment (Figure 1). However, no statistically significant difference was found between the two groups throughout the 6-week experimental period in terms of body weight (*P* > 0.05). In Group I, two rats died at 4 weeks after the start of the experiment, and in Group II, one died at 5 weeks after the start of the experiment.

### Behavioral studies

#### Assessment of sensory threshold values in hind paws

**Figure 2:**
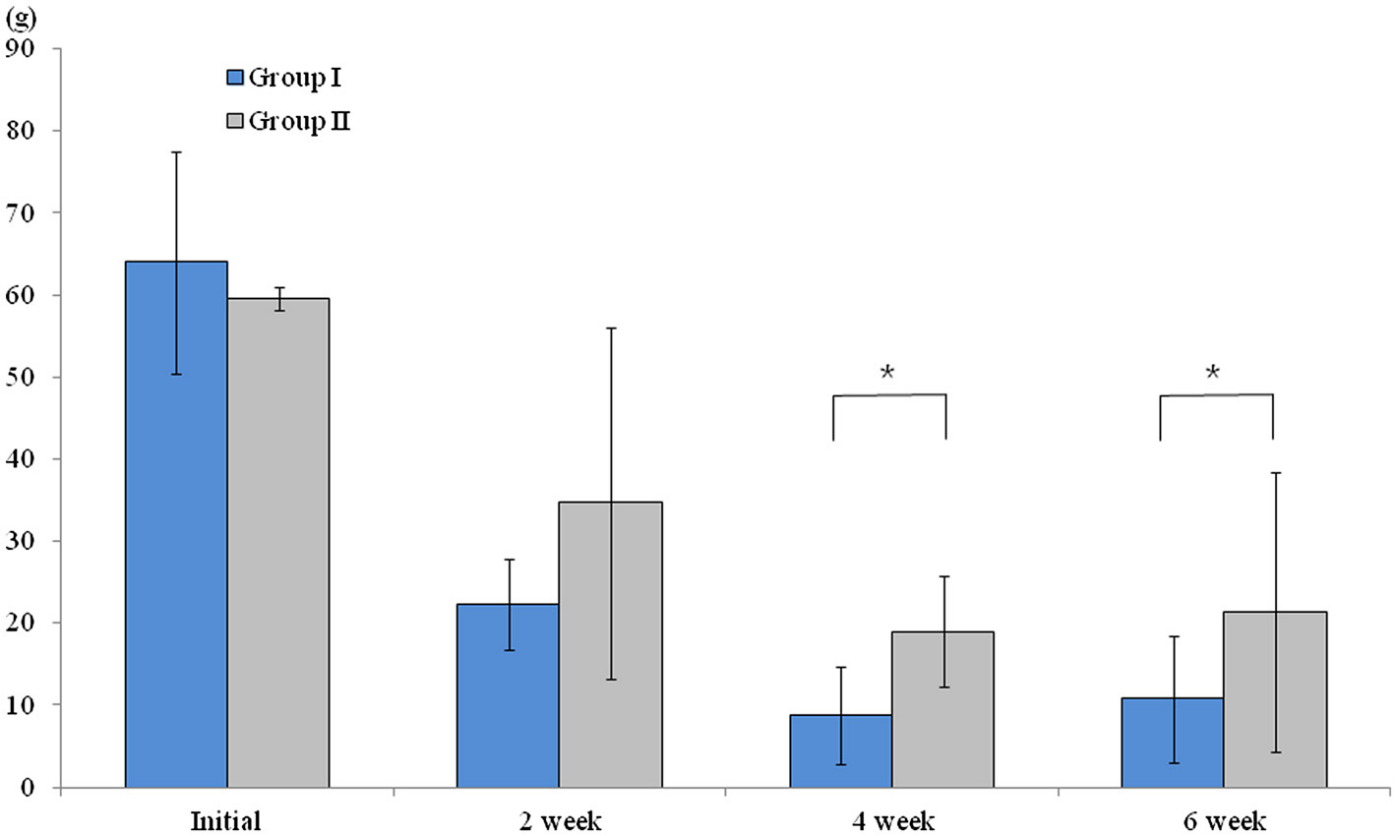
** The assessment of sensory thresholds using touch-test sensory evaluators.** Before and at 2 weeks after oxaliplatin administration, there was no significant difference in sensory thresholds between the two groups (*P > 0.05)*. However, at 4 and 6 weeks after oxaliplatin administration, the mean sensory thresholds of Group II with green tea(300 mg/kg orally once daily) were statistically higher than those of Group I without green tea(*P* < 0.05). * *P* < 0.05.

The sensory threshold test in the right hind paw was conducted before oxaliplatin administration and at weeks 2, 4, and 6 after oxaliplatin administration. Before oxaliplatin administration and at week 2 after oxaliplatin administration, there was no significant difference in sensory thresholds between the two groups (*P* = 1.0 and *P* = 0.39, respectively). However, at 4 and 6 weeks after oxaliplatin administration, a significant difference in mean sensory thresholds between the two groups was found; the mean sensory thresholds of Group II (19.9 ± 6.7 g and 20.6 ± 15.9 g, respectively) were higher than those of Group I (9.5 ± 5.9 g and 10.6 ± 7.2 g, respectively; *P* = 0.01 and *P* = 0.01, respectively; Figure 2).

#### Assessment of thermal threshold values in the tail

**Figure 3:**
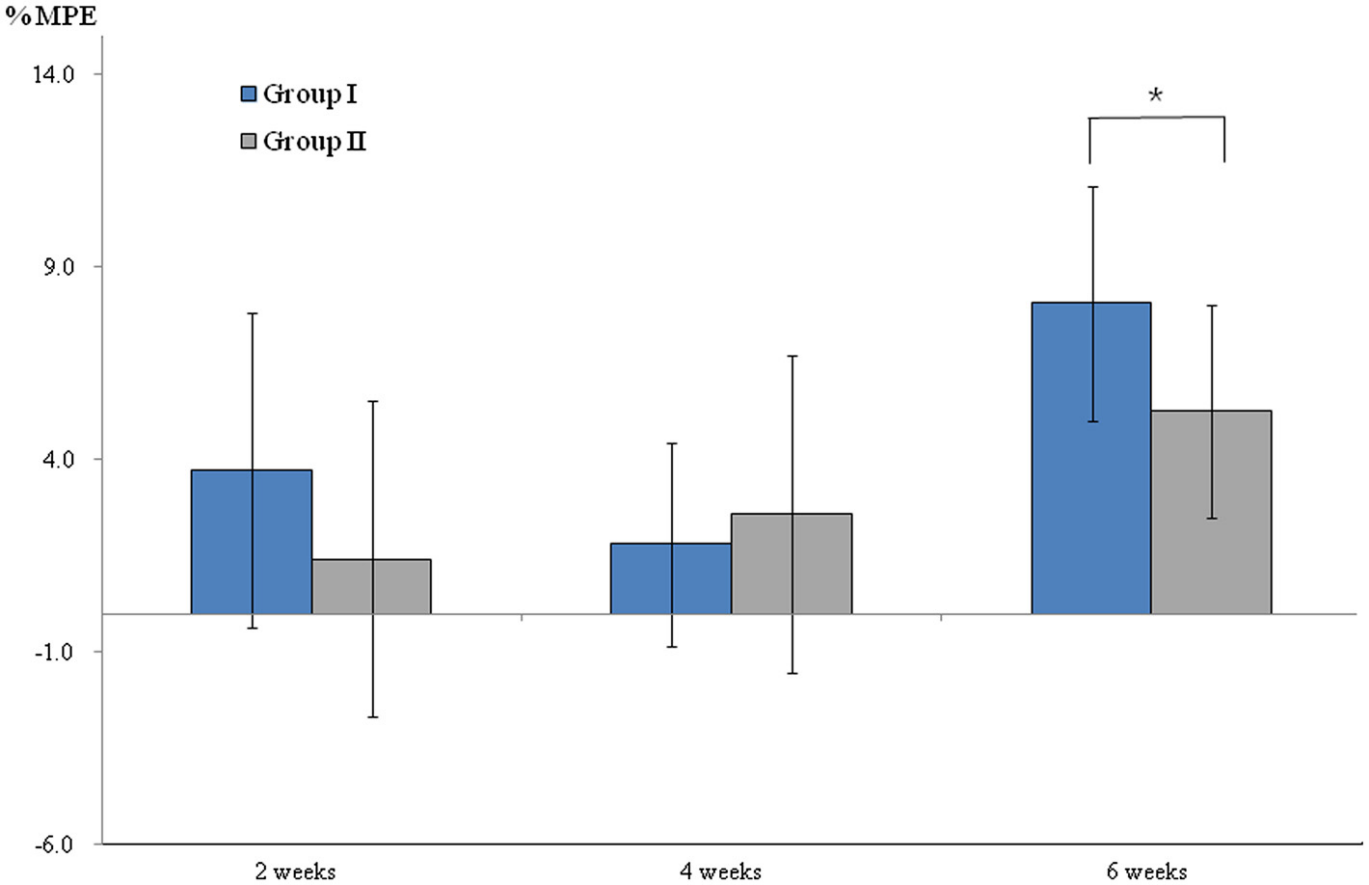
** The assessment of thermal thresholds using tail-flick analgesia meter.** Effects of green tea extracts on tail flick test after oxaliplatin administration indicated by maximal possible effect (%MPE) (mean ± SD), which showed the mean%MPE of Group II with green tea(300 mg/kg orally once daily) were statistically lower than those of Group I without green tea at 6 weeks after oxaliplatin administration (*P* < 0.05). * *P* < 0.05.

At 2 and 4 weeks after oxaliplatin administration, no significant difference was found in the mean% MPE between Groups I and II (*P* > 0.05). However, at 6 weeks after oxaliplatin administration, the mean% MPE of Groups I and II were 8.1 ± 3.0 and 5.2 ± 2.5, respectively, which showed the increased thermal threshold values in Group I when compared to the Group II taking green tea extracts (*P <* 0.05, Figure 3).

#### Neurophysiological evaluation

Sensory nerve conduction in the tail and the H-reflex-related SNCV were determined in all experimental animals at baseline and at 2, 4, and 6 weeks after oxaliplatin administration. At baseline, the peak latency, amplitude, and H-reflex-related SNCV were 2.9 ± 0.3 ms, 37.2 ± 10.6 μV, and 49.9 ± 10.3 m/s, respectively, in Group I and 2.9 ± 0.2 ms, 39.2 ± 10.0 μV, and 50.8 ± 6.7 m/s, respectively, in Group II; there was no significant difference between the two groups (*P* > 0.05). At 2, 4, and 6 weeks after oxaliplatin administration, no significant difference was observed in the mean peak latency, amplitude, or H-reflex-related SNCV between the two groups (*P* > 0.05, Tables 1 and 2).

**Table 1:** Sensory nerve conduction study in tail of rats

		**Initial**	**2 weeks**	**4 weeks**	**6 weeks**
Peak Latency (msec)	Group I	2.91 ± 0.27	2.96 ± 0.20	2.88 ± 0.20	2.76 ± 0.17
	Group II	2.94 ± 0.21	2.83 ± 0.22	2.74 ± 0.11	2.64 ± 0.14
Amplitude (μV)	Group I	37.16 ± 10.55	38.69 ± 6.29	37.77 ± 7.30	24.50 ± 6.81
	Group II	39.20 ± 9.96	32.90 ± 8.15	32.57 ± 13.96	23.69 ± 11.20

**Table 2:** H-reflex related sensory nerve conduction velocity in rats

	**Initial**	**2 weeks**	**4 weeks**	**6 weeks**
Group I	49.9 ± 10.3 m/sec	49.6 ± 7.3 m/sec	48.3 ± 6.7 m/sec	50.7 ± 8.2 m/sec
Group II	50.8 ± 6.7 m/sec	51.9 ± 12.5 m/sec	48.9 ± 13.8 m/sec	46.3 ± 12.8 m/sec

### TUNEL staining

**Figure 4:**
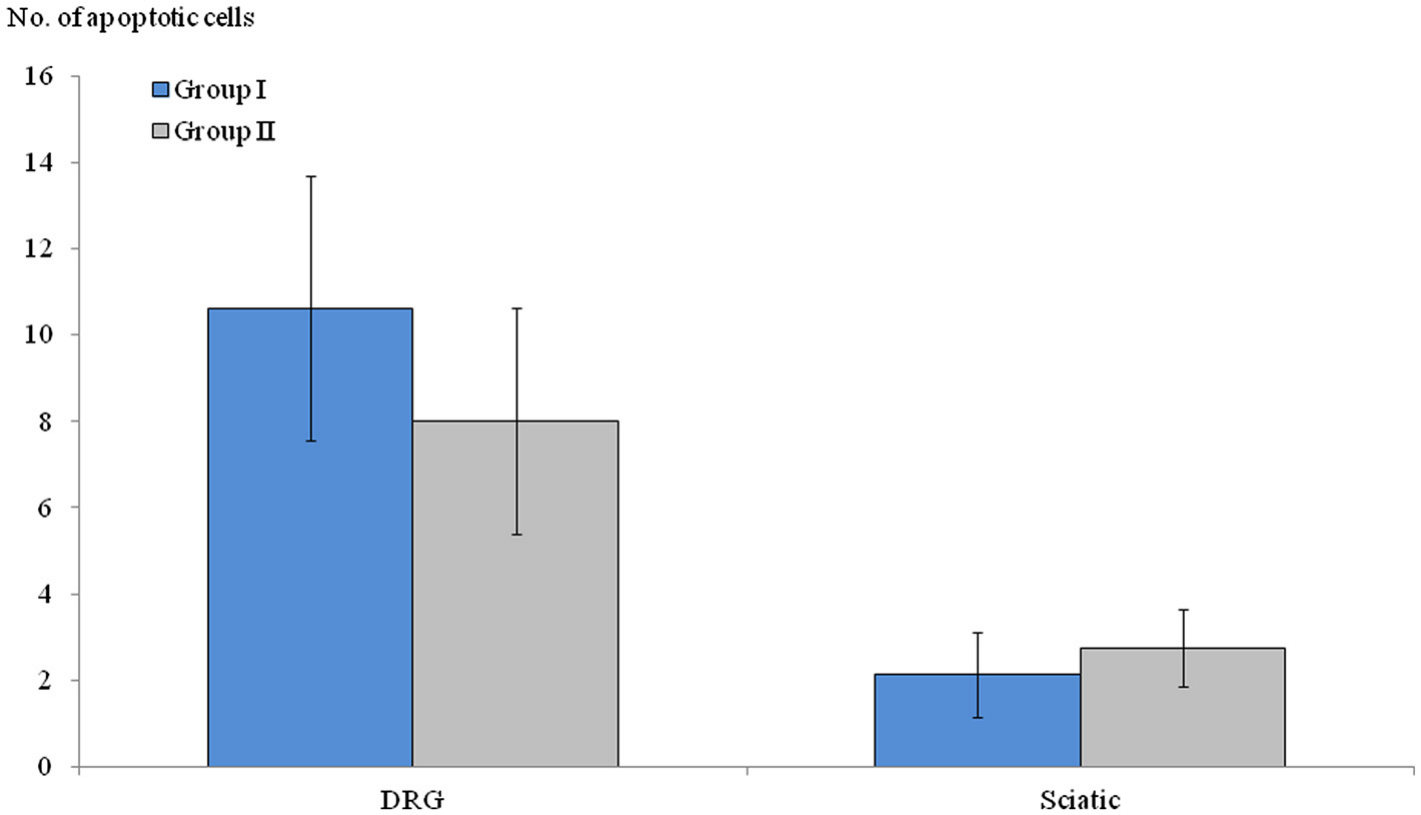
** The comparison of apoptotic-featured cells between the two groups.** The statistical significances of the number of apoptotic cells were not found in dorsal root ganglia or sciatic nerve between two groups, which were harvested at 6 weeks after oxaliplatin administration (*P >* 0.05).

**Figure 5:**
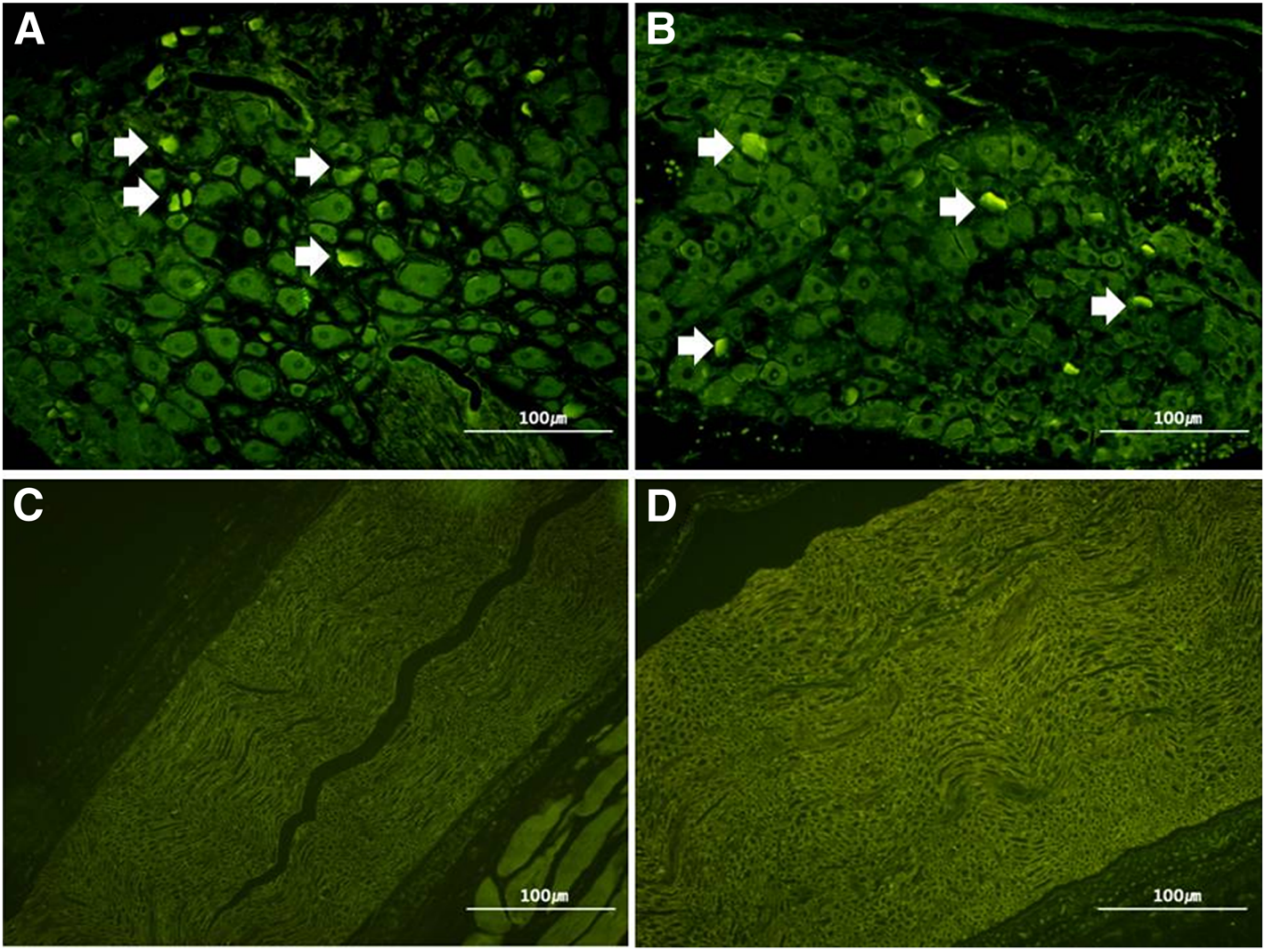
** The comparison of apoptotic-featured cells between DRG and sciatic nerve in both groups.** TUNEL staining shows the increased number of apoptotic-featured cells in dorsal root ganglia of two groups (**A**. Group I, **B**. Group II, White arrow), but apoptotic-featured cells were not easily found in sciatic nerve of two groups(**C**. Group I, **D**. Group II), x 200 magnification. The white arrow marks indicate the apoptotic-featured cells.

The mean numbers of apoptotic-featured cells in DRG and sciatic nerves were 10 ± 3 and 2 ± 1 in Group I and 8 ± 2 and 2 ± 1 in Group II, respectively; no statistically significant difference was seen between the two groups (*P* > 0.05, Figure 4). However, the number of apoptotic-featured cells in DRG was higher than that of sciatic nerves in Groups I and II (*P* = 0.01, Figure 5).

## Discussion

The present study investigated the preventive role of green tea extract, containing antioxidants, in oxaliplatin-induced PN using a rat model. The findings showed that green tea extracts alleviated mechanical allodynia or decreased thermal response after oxaliplatin administration, but did not prevent morphometric or electrophysiological alterations induced by oxaliplatin. Green tea extracts may induce a mild neuroprotective or palliative effect in a certain period time of sensory oxaliplatin-induced PN.

In this study, we observed that neurotoxicity symptoms in experimental Group II, which received oxaliplatin and green tea extracts, was significantly alleviated in the sensory threshold test using Von Frey hairs at 4 and 6 weeks (*P* = 0.01) and thermal threshold test at 6 weeks(*P* = 0.048) after oxaliplatin administration, compared with experimental Group I which received oxaliplatin alone. Based on the findings of this animal experiment, we hypothesize that the damaging properties of free radicals may have an effect for a certain period of time of oxaliplatin-induced sensory neuropathy. Findings reported by various investigators are consistent with this. Joseph et al. [16], in an experimental study with rat models, observed that oxaliplatin acts on nociceptors to induce oxidative stress-dependent acute peripheral sensory neuropathy, and that both systemic and local administration of antioxidants, such as acetyl-L-carnitine, alpha-lipoic acid, and vitamin C, markedly inhibited oxaliplatin-induced hyperalgesia. A recent report demonstrated that epigallocatechin gallate, a major polyphenolic catechin present in green tea, induces heme oxygenase-1 expression in cultured rat neurons, possibly by activation of the transcription factor Nrf2, and by this mechanism is able to protect against oxidative stress-induced cell death [17].

In the late course of oxaliplatin treatment, however, it continues to accumulate in nerve tissues and inevitably induces chronic toxicity. In this study, green tea extracts did not prevent morphometric or electrophysiological alterations by oxaliplatin. This indicates that a pathogenic mechanism other than free radical injury, including apoptosis of DRG cells [5], the impairment of cellular mitochondrial oxygen consumption [6], or prolonged activation of voltage-gated Na^+^ channels, which leads to excess Ca^2+^ influx [7], may also be significantly involved in chronic oxaliplatin-induced PN. Additionally, the relationship between acute manifestations and the development of chronic manifestations of oxaliplatin-induced PN has not been fully elucidated. A recent study reported that acute modulation of Na^+^ channel properties in both motor and sensory axons influences the final severity of oxaliplatin-induced PN [4]. Additionally, recent studies suggest that reactive oxygen species likely have a role in the link between acute and chronic manifestations [11].

No significant alteration in the nerve conduction study was found during oxaliplatin treatment, which may be explained by the fact that the main pathological changes in oxaliplatin-induced neuropathy occur predominantly in DRG[18] not sciatic nerves. Additionally, there was not enough to provoke histological alterations that could be detected as electrophysiological abnormalities in peripheral nerves, because electrophysiological parameters usually change with Wallerian degeneration of a given peripheral nerve. Contrary to our electrophysiological findings, an experimental animal study that evaluated the protective role of acetyl-l-carnitine in paclitaxel- and cisplatin-induced neurotoxicity showed somewhat different results: SNCV was significantly reduced compared with that in the control group in both cisplatin and cisplatin + acetyl-l-carnitine groups (*P <* 0.001 for both groups). The difference, however, was significantly less marked in the cisplatin + acetyl-l-carnitine group (control group (mean ± SD) 41.1 ± 3.0 m/s; cisplatin group 27.8 ± 1.9 m/s; and cisplatin + acetyl-l-carnitine group 33.4 ± 2.8 m/s), and the difference between the two groups of cisplatin-treated rats was significantly in favor of acetyl-l-carnitine cotreatment [19]. However, a study of oxaliplatin-induced neurotoxicity in humans showed that the most significant abnormality in the nerve conduction study results was a low-amplitude sensory nerve action potential (SNAP) in all eight symptomatic subjects. This reduction in sural SNAP amplitude was associated with a minor decrease in conduction velocity in symptomatic patients, although the mean sural nerve conduction velocity was not significantly altered [10]. These differing electrophysiological findings may be due to the temperature effect on the sensory nerve conduction study. All neurophysiological determinations were performed under standard conditions in a temperature-controlled room in an experimental animal study of reference articles [20], which caused critically false nerve conduction study results. The skin temperature, not the room or core temperature, is the most profound factor in nerve conduction studies. Because the general condition of animals that have received chemotherapy is continually aggravated, it is very difficult to maintain the skin temperature at an appropriate level (30 - 32°C) under standard conditions in a temperature-controlled room. Thus, lowering the temperature of a nerve has a direct effect on the nerve’s action-potential-generating mechanism at the nodes of Ranvier. Under these circumstances, the amplitude of the action potential is also increased, and the latency and conduction velocity are prolonged. The main change in nerve conduction study results in animal chemotherapeutic studies seems to be delayed latency and conduction velocity, not decreased amplitude. In this study, we used an infrared heat lamp to raise the surface temperatures of the tail, foot, and leg, to 29 - 30°C.

A nerve conduction study may be a useful tool for understanding the pathophysiology of neuropathies or for clinical monitoring of neurotoxicity in patients who undergo chemotherapy, but it is not thought to be a sensitive tool for acute oxaliplatin neurotoxicity, considering the technical differences between expert and non-expert practitioners. Further, the variability in the measurement of amplitudes of sensory action potentials is substantially greater than the variability in the measurement of amplitudes of motor action potentials. The conventional nerve conduction study lacks the sensitivity, while there is a report that sensory abnormalities in an axonal excitability test occurred prior to a significant reduction in the compound sensory amplitude and the development of neuropathy; additionally, excitability changes occurred before clinically significant neurotoxic symptoms. This suggests that assessment of sensory excitability parameters may provide a sensitive biomarker of severity for oxaliplatin-induced neurotoxicity [21].

Increasing interest in the oxidative stress induced by chemotherapeutic agents and its role in the mechanism of adverse effects has recently been addressed. To date, several antioxidant agents have been tested for their ability to prevent oxaliplatin-induced PN. In a randomized, double-blind, placebo controlled trial, glutathione effectively reduced oxaliplatin-induced PN clinically and electrophysiologically without reducing the clinical activity of oxaliplatin [22]. Alpha-lipoic acid has effectively reduced the severity of this dose-limiting oxaliplatin-related side effect in 15 patients with advanced colorectal cancer who were treated with an oxaliplatin-based regimen and experienced a ≥ grade 2 oxaliplatin-induced PN [23]. Pace et al. demonstrated that supplementation with vitamin E decreased the incidence (30.7% vs. 85.7%; *P* < 0.01) and severity (2 vs. 4.7; *P* < 0.01) of PN in a comparison of 27 patients receiving cisplatin chemotherapy with vitamin E with those receiving only cisplatin chemotherapy [15]. The effects of green tea on chemoprevention have been investigated. There is a report that administration of doxorubicin alone decreased tumor weight by 25% compared with the control level, whereas the addition of green tea markedly reduced the tumor weight, to 37% of the control level, and significantly enhanced the doxorubicin inhibitory effect on tumor growth, 2.5-fold. Green tea, theanine, and caffeine in combination with doxorubicin did not increase the doxorubicin concentration in the heart or liver of tumor-bearing mice [24]. Based on the fact that green tea, which has antioxidative properties, has a positive effect on cancer chemotherapy, we expect that the neurotoxicity of oxaliplatin could be minimized.

## Conclusions

In summary, considering that oxaliplatin administration produces an acute “functional” channelopathy of axonal Na^+^ channels, the findings of the present study suggest that green tea extracts seem to alleviate functional channelopathy, based on results at 4 and 6 weeks after oxaliplatin administration, although objective parameters such as electrophysiological and histological findings were apparently unaffected. Thus, even though they seem to have temporary effect on neurotoxicity, green tea extracts may be a useful adjuvant to alleviate sensory symptoms in the early stages of neurotoxicity in clinical settings.

## Competing interests

The authors have no conflicts of interest.

## Authors’ contributions

JSL participated in the design of the study, carried out the animal studies and performed the statistical analysis. WTK interpreted the electrophysiological study. EKJ carried out the immunohistochemical interpretive calibration. HSW carried out the immunohistochemical interpretive calibration and performed the statistical analysis. YSC participated in the design of the study and coordination. YHK participated in the design of the study, carried out the animal studies and prepared the article for publication. All authors read and approved the final manuscript.

## Pre-publication history

The pre-publication history for this paper can be accessed here:

http://www.biomedcentral.com/1472-6882/12/124/prepub
